# Strategic Management of Descemet’s Membrane Perforation During DALK in Advanced Keratoconus

**DOI:** 10.22336/rjo.2024.81

**Published:** 2024

**Authors:** Alina Gabriela Gheorghe, Ancuța Georgiana Onofrei, Ana-Maria Arghirescu, Andrei Coleașă, Georgia-Denisa Tiran, Laura Ioana Dinu, Elena Veronica Toader

**Affiliations:** 1 “Carol Davila” University of Medicine and Pharmacy, Bucharest, Romania; 2 Department of Ophthalmology, Clinical Hospital for Ophthalmological Emergencies Bucharest, Bucharest, Romania

**Keywords:** Deep Anterior Lamellar Keratoplasty (DALK), recovery techniques in DALK, postoperative management of Anterior Lamellar Keratoplasty, outcomes of Anterior Lamellar Keratoplasty, Keratoconus (KC), Intraoperative Anterior Segment OCT, AC =Anterior Chamber, AI =Artificial intelligence, AS-OCT = Anterior segment ocular coherence tomography, BB = Big Bubble, CAIRS = Corneal Allogenic Intrastromal Ring Segments, CXL = Corneal Cross-Linking, DALK = Deep anterior lamellar keratoplasty, dDALK = descemetic DALK, Deg = Degree, DL = Dua’s layer, DM = Descemet’s membrane, DMEK = Descemet Membrane Endothelial Keratoplasty, ICRS = Intracorneal Stromal Ring Segment, IOP = Intraocular Pressure, K-Max = Maximum Keratometry, KC = Keratoconus, KCE = Keratoconus enlargement, PK = Penetrating keratoplasty, RGP = Rigid Gas Permeable Lenses

## Abstract

**Objective:**

To report on the surgical treatment of advanced keratoconus (KC) with stromal scarring in a young male patient with asymmetric disease progression complicated by an intraoperative microperforation of Descemet’s membrane (DM) during deep anterior lamellar keratoplasty (DALK).

**Methods:**

The surgical approach consisted of manual descemetic DALK (dDALK), further complicated with DM microperforation. Anterior segment ocular coherence tomography (AS-OCT) was used intraoperatively to locate the site and size of the tear. The surgeon decided not to convert to penetrating keratoplasty (PK), despite stromal scarring, significant ectasia, and variable corneal thickness, but rather to continue the dissection of the stromal bed with maximum precaution.

**Results:**

Postoperatively, visual results improved and reached the best corrected visual acuity of 20/20. Choosing a proper graft dimension and reaching anatomical separation up to the DM were the keys to obtaining such a positive refractive outcome.

**Discussions:**

DALK, the most advanced treatment for KC, was chosen as the ideal option for this young patient due to its advantages over PK: reduced rejection risk, fewer complications, quicker steroid tapering, and faster recovery. However, its steep learning curve remains a challenge for surgeons.

**Conclusions:**

Despite manual DALK being a more challenging and time-consuming procedure than PK, careful dissection of the stromal bed and diligent assessment of the affected DM can provide a better and safer outcome for selected patients. Even if initial postoperative visual results are impressive, the surgeon must pay attention to the patient’s future check-ups to swiftly correct any possible complications.

## Introduction

Keratoconus (KC), the most common corneal ectasia worldwide, is characterized by progressive thinning and conical protrusion of the cornea that can lead to significant visual impairment if the appropriate treatment is not applied [1]. The incidence is rising worldwide, driven by a genuine increase in cases and improvements in clinical and diagnostic technologies that facilitate earlier and more precise diagnoses [1,2].

The human cornea consists of the epithelium and Bowman’s layer at the front and the endothelium with its basement membrane, known as Descemet’s membrane (DM), at the back, with the stroma in between. Because of the differences in proteoglycan composition, the anterior stroma is thicker than the posterior [3].

Depending on the stage of KC found at the first presentation, different detection and treatment methods are expected. Four stages of keratoconus were described: subclinical, early, moderate, and advanced. These stages are based on clinical presentation and topography [4].

In subclinical cases, the mandatory signs are normal visual acuity, no unusual biomicroscopic findings, and modified tomography (abnormal localized steepening or asymmetric bow-tie pattern).

Besides these, at least another criteria from the following must be met: CCT < 500 micrometers, K power > 47.00 D, oblique cylinder > 1.5 D, or clinical signs of KC in fellow eye [5].

More recently, ophthalmic devices based on combined corneal tomography techniques (“scanning slit”, Scheimpflug, anterior segment optical coherence tomography) include KC screening tools based on artificial intelligence (AI). The modified corneal architecture in KC patients can be studied on various computerized maps, such as curvature keratometric, elevation, thickness, total corneal power, and epithelial thickness [6].

Finally, yet importantly, the epithelial thickness profile is beneficial for detecting and characterizing keratoconus eyes. A potential marker for keratoconus is a distinctive doughnut-shaped pattern in the epithelium that usually indicates the presence of an underlying stromal cone. However, in the early stages of the disease, epithelial compensation may mask the presence of the cone, making detection more challenging [7]. In KC, basal epithelial density decreases, and basal epithelial layers degenerate. It is essential to exclude other corneal epithelial alterations like dry eye, high myopia, contact lens use, prior refractive surgery, etc. [8].

KC is also characterized by histological changes in the normally acellular Bowman’s layer. Cellular elements have been observed, with several breaks distinguished within this layer. Additionally, proliferative collagenous tissue from the anterior stroma can be detected in keratoconic corneas [9].

Further studies have also identified cytohistological changes in failed corneal grafts with a KC history. Bowman’s layer ruptures appear more common than corneas without underlying KC, typically occurring five years after corneal transplantation [10].

Moving forward, the following stages of KC, early and mild ones, bring changes such as a reduction in visual acuity, some differences in refractive error along with significant aspects in tomographic imaging (more evident corneal steepening and thinning, keratometric differences between the superior and inferior cornea, corneal aberrations). The oil droplet reflex can be seen in this stage while performing direct ophthalmoscopy [4].

Furthermore, corneal scarring, measurement artifacts, edema, and other abnormal corneal factors can also lead to a false positive diagnosis of KC [11]. Therefore, conducting multiple imaging techniques to detect this disease is essential.

Progression of KC will reduce visual acuity, cause rapid changes in refractive readings, increase keratometric differences between the superior and inferior cornea, and cause mild corneal steepening and thinning [4].

Studies show that patients with progressive keratoconus tend to be younger than nonprogressive cases, with pediatric cases showing faster progression. Key risk factors for children include a corneal thickness below 450 micrometers, mean K readings over 50 D, and increased posterior elevation [5]. Evaluation of corneal topography is considered the best method for early detection and monitoring of keratoconus progression, and the Pentacam tomography system is the most sensitive method. Changes in keratoconic corneas are very complex. Efforts have been made to find the most reliable variables to explain progression, but most reflect changes in the central cornea or the steepest point in corneal curvature. However, it is possible to document changes in the cornea’s curvature out of the steepest point. Despite not meeting progression criteria such as Kmax or pachymetry, some patients had changes in the anterior curvature maps that could mean progression. Thus, we define keratoconus enlargement (KCE) as an increase of more than 1D in the anterior corneal curvature of the non-apical area out of the steepest point Kmax represents [12].

Annual increases in keratometry of 1.0 to 1.5 diopters and changes in corneal elevation indicate progression. Corneal thickness below 350 micrometers and a 2% yearly decrease in central corneal thickness are linked to disease advancement. Changes in corneal astigmatism are also strong predictors of progression. Corneal biomechanical properties, modeled through advanced simulations, provide additional insight, and a higher neutrophil-to-lymphocyte ratio has been associated with progressive keratoconus. Family history is another important factor, as genetic predisposition increases the risk, making it crucial to screen relatives for potential development of the condition [5].

Advanced KC can be detected by performing a slit lamp examination and looking for the following clinical signs: Vogt’s striae, Munson sign, Fleischer ring, severe thinning and steepening (with dioptric values over 55 D), and corneal thinnest point around 300 µm with visual acuity under 20/400 (< 0.05) [13,4].

During the first stages of KC, non-surgical treatment options should be discussed. Spectacles are generally well tolerated and can correct slight refractive and visual acuity errors. However, when the disease progresses, it will not be enough to compensate for the loss of vision. Thus, contact lens wear is required for visual recovery. Various contact lens options are available, including rigid gas permeable lenses (RGP), soft or soft toric lenses, bicurved hard lenses, hybrid lenses, and piggyback system lenses. Although most keratoconic patients will need RGP lenses, some patients, especially those with minimal disease, may have better vision outcomes and a more comfortable wear experience with soft lenses. Keratoconic patients without central scarring and contact lens intolerance with mild to moderate disease may be contenders for intracorneal stromal ring segment (ICRS) insertion [14-16].

The surgical management of advanced keratoconus has recently seen valuable progression, with DALK successfully replacing PK. DALK avoids the risks induced by PK, such as endothelial rejection and the complications associated with “open sky” surgery (endophthalmitis and expulsive hemorrhage). DALK is the treatment of choice in cases of corneal pathologies involving the epithelium, Bowman’s membrane, or stroma, as long as the endothelium remains functional[17]. It was once thought that techniques like the Big Bubble (BB) method and mechanical tools fully exposed the DM during DALK. However, recent studies have revealed the presence of residual stroma on the DM in some cases. DALK and ultrathin Descemet’s stripping endothelial keratoplasty have fewer interface complications than other corneal surgeries [3].

The most common indications for DALK are KC and corneal stromal scarring. The intraoperative complication specific to the DALK technique is DM perforation. The risk of perforating the DM is increased with an irregular, thin cornea preoperatively and with high intraocular pressure [18].

The presence of a DM microperforation (less than 1/4 of the cornea) is no longer considered a criterion for DALK to PK conversion [19]. Among corneal pathologies, KC is known to be predisposed to DM perforation or rupture [20].

## Case presentation

We report the case of an 18-year-old male patient with a history of asymmetric bilateral KC who sought treatment at our clinic due to a progressive decline of visual acuity and photophobia. He had previously been diagnosed with stage 2 KC in the right eye and stage 4 KC in the left eye. No other systemic diseases were reported.

Upon examination, the best corrected visual acuity was measured at 0.9 in the right eye and counting fingers in the left eye. Non-contact tonometry indicated normal tension in both eyes.

**Fig. 1:**
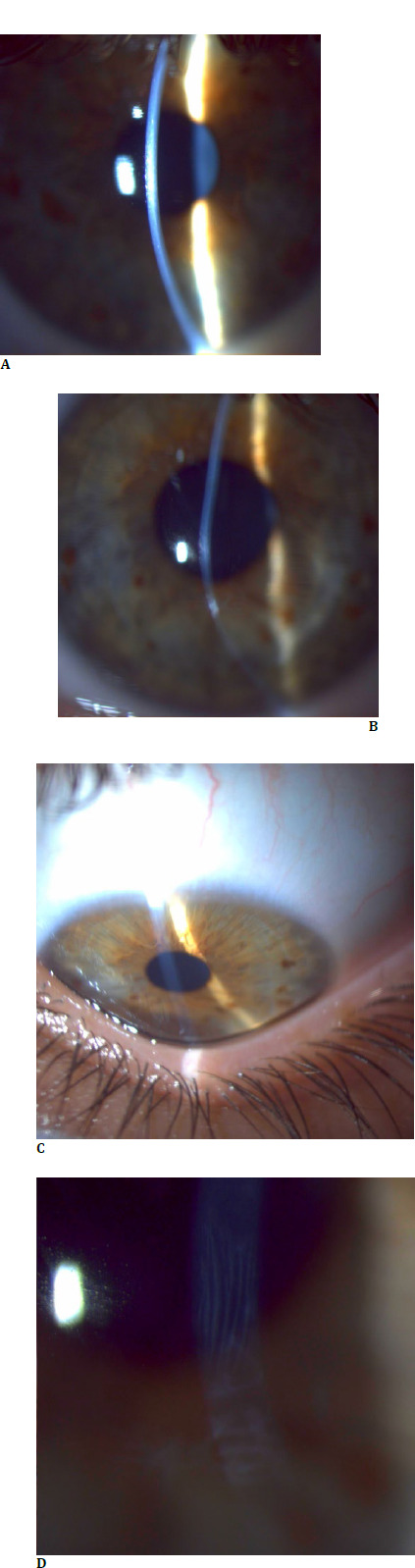
Clinical findings: Right eye - (**A**) mild scarring after CXL; Left eye - (**B**) inferior paracentral corneal ectasia; (**C**) Munson sign; (**D**) Vogt’s striae (personal photo archive of Alina Gheorghe, MD, PhD)

The slit lamp examination revealed mild paracentral corneal ectasia, with slight stromal scarring following the CXL procedure in the right eye. The left eye exhibited paracentral corneal ectasia, a Fleischer ring, superficial stromal scarring, and a deep anterior chamber. Both Munson’s and Rizzuti’s signs were positive. Fundoscopic examination did not reveal any pathological findings.

**Fig. 2:**
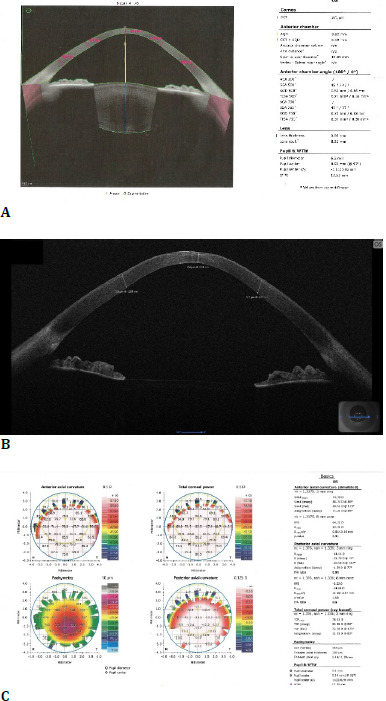
Left eye: (**A**)+(**B**) AS-OCT showing decreased central corneal thickness and superficial stromal fibrosis; (**C**) stage IV keratoconus according to the ABCD classification (personal photo archive of Alina Gheorghe, MD, PhD)

Corneal topography of the left eye showed modified parameters, with a mean simK 74D and a thinnest pachymetry measurement of 354 µm. Superficial stromal scarring and variable corneal thickness were visible on AS-OCT.

**Fig. 3:**
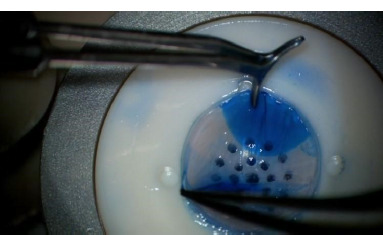
Graft preparation: Descemet’s membrane and endothelium are removed (personal photo archive of Alina Gheorghe, MD, PhD)

The patient underwent manual DALK following corneal thickness map results. The surgeon prepared the graft using the liquid bubble dissection technique to use the remaining endothelial and Descemet lenticule for DMEK surgery. A partial bubble was obtained (**Fig. 3**). After trephination, the Descemet endothelial lenticule was manually harvested with the remaining stroma for DALK.

Partial trephination of about ⅔rd of the total corneal thickness was conducted using an adjustable-depth vacuum trephine. Stromal removal was achieved using a crescent blade and was followed by layer-by-layer stromal dissection until we approached the descemetic membrane. Intraoperative OCT was used and proved to be a valuable tool for visualizing the size of the residual stromal bed.

**Fig. 4:**
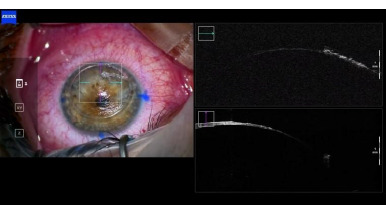
Intraoperative OCT (personal photo archive of Alina Gheorghe, MD, PhD)

During dissection of the deeper corneal layer, due to direct exposure of the Descemet membrane, a small peripheric microperforation occurred. Rescue techniques were applied, and conversion to PK was avoided by lowering the IOP through paracentesis, inserting air in the AC, and carefully dissecting the overlying stromal tissue.

**Fig. 5:**
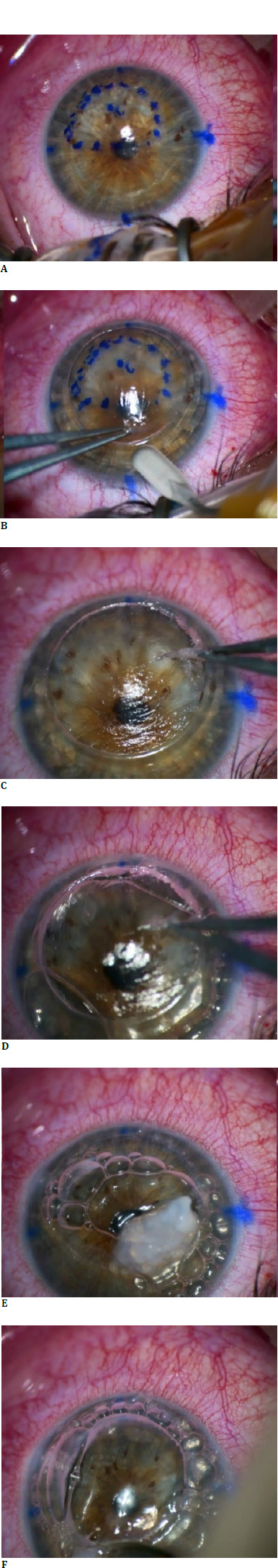
Surgical steps: (**A**) marking of the corneal ectasia; (**B**) trephination and dissection of the corneal lamellae; (**C**) observing the Descemet perforation; (**D**) visualizing the posterior corneal surface by injecting air into the anterior chamber; (**E**) resuming stromal dissection; (**F**) Descemet layer exposed; (personal photo archive of Alina Gheorghe, MD, PhD)

**Fig. 6:**
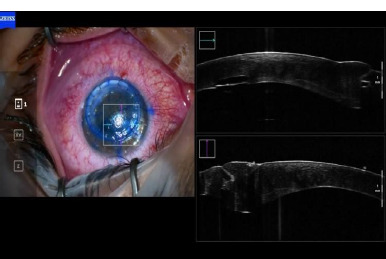
Final intraoperative look (personal photo archive of Alina Gheorghe, MD, PhD)

At one week follow-up, the best corrected visual acuity was 0.6. The autorefractometry results were -0,5dsf -1.75 cyl 50 deg.

**Fig. 7:**
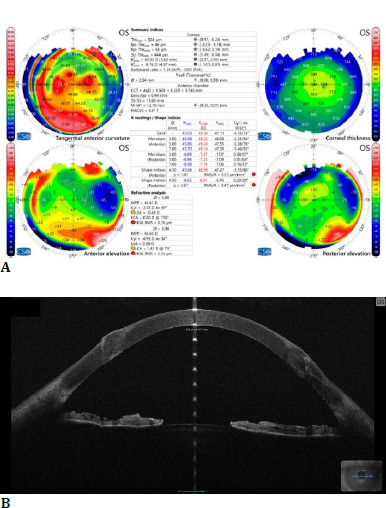
Three months follow-up: (**A**) topography showing mild inferior ectasia; (**B**) AS-OCT image, central corneal thickness 508 µm (personal photo archive of Alina Gheorghe, MD, PhD)

At the 3-month follow-up, inferior suture repositioning was required due to decreased visual acuity caused by subsequent suture laxity. A modified topographical map, which revealed mild inferior ectasia, corroborated this.

**Fig. 8:**
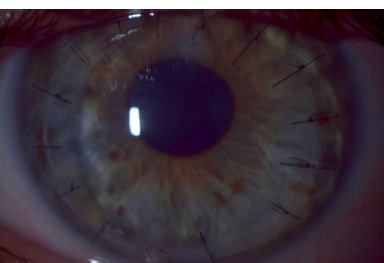
Left eye, 3 months post DALK surgery (personal photo archive of Alina Gheorghe, MD, PhD)

One month post-repositioning, autorefractometry results were: -1.5 dsf -4d cyl 120 deg, with a maximum keratometry (K-max) of 48 diopters, and BCVA increased to 1.

## Discussions

It is worth considering that early diagnosis is the key to success for keratoconic patients. It allows the cornea specialist to intervene as quickly as possible to slow down the progression of the disease by using different types of conservative treatments, such as soft or rigid contact lenses, CXL, ICRS, and CAIRS [21].

The patient underwent CXL in both eyes. It was effective in stabilizing the disease’s progression in the right eye. However, the left eye was no longer eligible for conservative treatment options, so different keratoplasty techniques were considered to restore the cornea's curvature.

With DALK being the cutting-edge treatment for KC, the surgeon considered it to be the optimal surgical approach for this young patient, taking into consideration the number of advantages the technique has compared to PK: decreases the risk of transplant rejection by preserving the endothelium; has fewer intraoperative complications, the procedure is done without having to open the eye completely; offers the possibility of steroid tapering more quickly; allows for faster healing time. Conversely, performing DALK comes with considerable struggles for corneal surgeons, as it comes with a longer learning curve.

Obliterating the patient’s stroma remains the main intraoperative challenge. The visual outcome depends on the regularity of the interface between the donor stromal graft and the host’s DM [21].

Retaining Dua’s layer (DL) alongside the DM and endothelium during DALK does not seem to increase the risk of interface haze. The residual stroma, including the DL, is less than 100 μm but usually around 80 μm, with good visual outcomes, similar to DMEK results. It is also worth remembering that while the anterior layers mainly support the cornea structure, the DL may also contribute to its biomechanical stability. Thus, understanding the DL is essential for performing corneal surgery and fully understanding conditions like KC and pre-Descemet localized dystrophies [3].

Our approach to manual DALK was to groove, dissect, and dissect. It is a consecrated technique and is reported as safe for cases like our patients’ (corneas are too thin and distorted). Precautionary measures were taken to dissect the stroma and preserve the healthy endothelium safely. However, a DM microperforation occurred. Because the perforation site was small and peripheral, it was decided to continue the procedure rather than convert to PK.

The surgeon must always be prepared for unexpected intraoperative complications, such as perforation of the DM. It can occur with any surgical DALK technique and during any step. This happens especially in dDALK. The rescue techniques consisted of paracentesis to lower the intraocular pressure to prevent enlargement of the perforation site, intracameral air tamponade used as a reference to evaluate the depth of host stromal dissection, and carefully resuming the dissection away from the perforation site [22]. Intraoperative DM perforation is a surgical challenge, and the management of these cases remains a disputed topic.

Part of the irregular stroma should be left in situ in a DM perforation to avoid spreading the tear. Additionally, the AC should be maintained by inserting air or viscoelastic substances during the dissection phase. Air or gas seals the rupture zone to close the DM perforation and is kept in the AC for a few days. During this period, careful patient monitoring is mandatory to handle any possible angle closure [23] promptly.

Many papers studied the visual outcome, graft failure, risk of endothelial decompensation, and postoperative complications of DALK with intact DM vs. DALK with micro/macro-perforation. They showed an increased risk of endothelial decompensation within complicated DALK, but no statistically significant differences were found regarding other elements between those two groups [24-26].

DM perforation can occur at any time during the procedure, but it most frequently occurs during deep lamellar dissection [17], as in our case.

Even if, in theory, the “best treatment” for DM perforation is not to reach this point in the first place, in practice, this cannot always be achieved. Knowing multiple rescue techniques is mandatory for any corneal surgeon. Using adjustable depth trephines, applying intraoperative pachymetry, lowering the AC pressure, performing paracentesis very peripheric, gentle air injection, and dissecting beyond the donor bed diameter are some of the rescue techniques applied by corneal surgeons worldwide.

The conversion rate to PK varies between 0.37% and 60%, mainly based on the surgeon’s experience [27].

DM perforation is known to be the main intraoperative complication (up to 57% of all complications). This can vary based on the diagnosis, the surgical technique, and the surgeon’s experience. Some risk factors for DM perforation are deep corneal stromal scarring, extreme thinning of the cornea, advanced corneal ectasia, and sharp instruments used for lamellar dissection [27]. DALK patients need regular check-ups to maintain good visual outcomes and to detect and treat any possible complications that can arise, including the “double anterior chamber” phenomenon (often observed in DALK patients with DM perforation and air bubble in the AC), graft failure (lower incidence than PKs), increased intraocular pressure (due to steroids drops), complications associated with sutures (loose suture thread etc.), recurrence of hereditary stromal dystrophies, and steroid-induced cataract [28].

One of the postoperative complications encountered was the loosening of corneal sutures. This is a relatively frequent complication observed at follow-ups. We documented it using topographical maps. Following this, surgical measures were taken to reposition the running sutures. Overall, results remain good to this day.

Suture-related complications are common during the postoperative period of DALK. Loose sutures need to be replaced to prevent possible infections and inflammation. Such adjustments can be made anytime without damaging the adhesion between the donor graft and the patient’s DM [23].

Researchers are trying to develop new treatment methods, such as implantation of intrastromal fresh lenticules, gene-based treatment, or less invasive CXL through intact epithelium [29].

KC should be made more aware so patients can take advantage of less invasive treatment options. Other comorbidities should be remembered, and questions about eye rubbing, allergy, eczema, and systemic diseases should be considered [30].

## Conclusion

Mastering all DALK techniques is a significant advantage for corneal surgeons. It allows them to re-evaluate and properly handle intraoperative issues while postponing the conversion to PK. DALK demands a long and laborious learning curve to obtain successful outcomes. All efforts not to convert to PK bring lower rates of complications, but this decision should be made based on the surgeon’s experience. Moreover, while visual recovery can be excellent initially, it is essential to regularly assess the healing process to manage any possible complications quickly. This allows for better refractive results, which is crucial for any patient, especially for one as young as ours.
